# Differences of lung microbiome in patients with clinically stable and exacerbated bronchiectasis

**DOI:** 10.1371/journal.pone.0183553

**Published:** 2017-08-22

**Authors:** Min Kwang Byun, Joon Chang, Hyung Jung Kim, Seok Hoon Jeong

**Affiliations:** 1 Division of Pulmonology, Department of Internal Medicine, Gangnam Severance Hospital, Yonsei University College of Medicine, Seoul, Korea; 2 Division of Pulmonology, Department of Internal Medicine, Severance Hospital, Yonsei University College of Medicine, Seoul, Korea; 3 Department of Laboratory Medicine and Research Institute of Bacterial Resistance, Gangnam Severance Hospital, Yonsei University College of Medicine, Seoul, Korea; Lee Kong Chian School of Medicine, SINGAPORE

## Abstract

**Background:**

Molecular-based diagnostic techniques can compensate for the inherent limitations of culture-based microbiology and provide a more comprehensive description of an entire community of bacteria at a particular anatomical site. Using culture-independent DNA-based molecular techniques, the aim of the present study was to characterize, differentiate, and compare the composition of lower airway bacterial microbiome between clinically stable and acutely infected patients with bronchiectasis experiencing exacerbation.

**Methods:**

Patients with clinically stable bronchiectasis and those experiencing acutely exacerbated bronchiectasis were recruited. All patients underwent bronchoscopy. Paired sputum and bronchoalveolar lavage (BAL) samples were collected for microbiological tests. Molecular analysis was performed for BAL samples using 16S ribosomal RNA (rRNA) gene sequencing.

**Results:**

The mean age of the 14 recruited patients was 60 years (range 42 to 78 years), and nine (64%) were female. Using quantitative culture and 16S rRNA sequencing, the common organisms identified from 14 BAL samples were *Haemophilus influenzae*, *Pseudomonas aeruginosa* and *Moraxella catarrhalis*, and *Prevotella*. Molecular techniques revealed *Prevotella* and *Veillonella* as potentially pathogenic anaerobic species. 16S rRNA gene sequencing yielded similar relative abundances and distributions of taxa in the stable and exacerbated bronchiectasis groups. Alpha diversity with richness, Simpson’s and Shannon indices, and beta diversity using principal coordinate analysis revealed no significant differences in lung microbiome between patients with clinically stable and exacerbated bronchiectasis.

**Conclusion:**

Culture-based microbiological and molecular-based techniques did not reveal significant differences in the lung microbiome of patients who were clinically stable and those experiencing exacerbated bronchiectasis. Patient-specific microbial communities were dominated by one or several genera, regardless of clinical status. DNA sequencing could identify potentially pathogenic organisms unable to be identified using microbiological methods.

## Introduction

Non-cystic fibrosis bronchiectasis (simply referred to in this report as bronchiectasis) is a chronic disorder of the bronchi and bronchioles. It is characterized by destruction and abnormal dilatation of the large airways [1]. As a consequence of the associated dysfunction of mucociliary clearance, a vicious cycle is established involving persistent bacterial colonization, chronic inflammation of the bronchial mucosa, and progressive parenchymal destruction [2]. Patients with bronchiectasis frequently experience acute exacerbation characterized by symptoms of fever, productive cough, mucopurulent sputum, and progressive dyspnea [3].

Although there are many causes of bronchiectasis, the most common is idiopathic. Several bacterial species are associated with lower airway infection in bronchiectasis. Traditional culture-based methods have identified *Pseudomonas aeruginosa*, *Haemophilus influenzae*, *Streptococcus pneumoniae*, *Staphylococcus aureus*, and *Moraxella catarrhalis* [4]. *P aeruginosa* is associated with accelerated decline in lung function in individuals with cystic fibrosis (CF) and has a detrimental effect on prognosis in bronchiectasis [5,6].

Management of bronchiectasis requires treatment of the underlying causes and control of recurrent infection. Recent management strategies emphasize the importance of bronchial hygiene, and the reduction of bronchial inflammation using inhaled corticosteroids and antibiotics [7].

Clarifying the role of bacterial infection in chronic disease and acute exacerbation is important in understanding the relationship between lower airway bacterial species and bronchiectasis. Current culture-based diagnostic microbiology techniques have limitations because certain bacterial species are refractory to growth under standard conditions and are exceedingly difficult to identify. The application of culture-independent techniques, including next-generation sequencing, have enabled the detailed characterization of lower airway bacterial communities and have revealed significantly greater bacterial diversity than previously reported methods using standard diagnostic microbiology [8–11].

Recently developed molecular techniques provide a comprehensive description of an entire community of bacteria at a particular anatomical site. Despite the importance of the microbe-host relationship in the pathogenesis and progression of infectious diseases, these approaches have not been applied to define the lung microbiome in patients with bronchiectasis.

The aim of this study, therefore, was to characterize, differentiate, and compare the composition of lower airway bacterial microbiome between clinically stable patients with bronchiectasis and acutely infected patients with bronchiectasis experiencing an exacerbation, using culture-independent, DNA-based molecular techniques. Comparing bacterial diversity and abundance between patients with stable and acutely exacerbated bronchiectasis may lead to improved understanding of the clinical significance of acute exacerbations in patients with bronchiectasis. Furthermore, these data may support the development of antimicrobial therapy guidelines to ensure effective treatment strategies for patients infected with colonizing bacteria.

## Materials and methods

### Patients

Patients with bronchiectasis confirmed by high-resolution computed tomography within the past 3 years, who attended the pulmonology outpatient clinic at Gangnam Severance Hospital (Seoul, Korea) between January and December 2016, were considered eligible. Investigators explained the aim and protocol of this study to patients who met the inclusion criteria. Informed written consent and medical history were obtained at the visit. Patients with bronchiectasis were recruited during a stable phase of their disease, defined as no exacerbation or antibiotic treatment in the previous 4 weeks. Patients with acutely exacerbated bronchiectasis, defined according to the criteria reported by Fuchs et al [12], were recruited as the comparator group. For all patients, sputum and bronchoalveolar lavage (BAL) samples were collected before initiation of antibiotics. Subjects were enrolled if they were adults (>19 years of age). No patients had a history of asthma or atopy, recent respiratory tract infection (within 4 weeks), or conditions with the potential to affect the safe performance of bronchoscopy. Patients with bronchiectasis had no evidence of (non-bronchiectasis) acute or chronic respiratory disease and were not receiving systemic corticosteroids. All subjects were macrolide naïve and none received nebulized antibiotics. Exclusion criteria included the following: (patients with) bronchiectasis as a result of focal endobronchial obstruction; currently active tuberculosis or non-tuberculous mycobacterium infection; recent deterioration in other respiratory disease(s); prescribed either oral or intravenous antibiotics therapy in the preceding 4 weeks; active malignancy; and acute cardiac disease.

This study was performed in accordance with the Declaration of Helsinki and Korean Good Clinical Practice guidelines. All protocols were approved by the institutional review board of Gangnam Severance Hospital (IRB #3-2016-0104).

### Clinical data

Demographic information (age, sex) and clinical data (lung function, serum inflammatory markers, and BAL fluid analysis) were recorded.

### Bacterial isolation and detection

All bacteria detected were quantified according to total viable count in the sputum (colony-forming units per milliliter of sputum [cfu/ml]). BAL fluid was incubated for 72 h after collection. Bacteria were identified and reported quantitatively, whether they grew >10^4^ cfu/mL or grew <10^4^ cfu/mL, but were identified as a single Gram-negative bacillus that was the only reportable pathogen. Another part of the BAL sample was used for polymerase chain reaction (PCR) and Sanger sequencing of 16S ribosomal RNA (rRNA) genes.

### Lower airway sample collection

Spontaneously expectorated sputum sample was collected from each patient, transported to the microbiology laboratory, and immediately processed for bacterial culture. All patients simultaneously underwent bronchoscopy under conscious sedation. After the bronchoscope was successfully wedged in the bronchus and bronchiectasis was confirmed in chest computed tomography scans, BAL fluid was collected from two or more segments in the dependent lesion. Warm sterile saline (150 mL) was gently instilled, followed by gentle aspiration through the bronchoscope’s suction channel. After the procedure, patients were observed for 2 h before being sent home. All BAL samples were divided into two separate bottles: one sample was processed for qualitative and quantitative cultures; the other was frozen at -80°C within 1 h for subsequent molecular analysis. Molecular analysis was performed on BAL samples only.

### DNA extraction

For all BAL samples, DNA was extracted using a commercially available DNA extraction kit (MG blood Genomic DNA Extraction SV kit, MGmed, Seoul, Korea) according to the manufacturer’s protocol. Briefly, a BAL sample (kept on dry ice) was transferred to an Eppendorf tube and weighed. Approximately 20 mL of BAL sample was vortexed thoroughly until homogenization was complete. DNA quality was determined using spectrophotometry (Qubit® 2.0 Fluorometer, Invitrogen, Eugene, OR, USA). Extracted DNA was stored at -20°C until use for polymerase chain reaction (PCR). Full details of the methodology used are provided in the S1 File.

### 16S rRNA PCR amplification and sequencing

For all BAL samples, DNA sequencing was performed using a commercially available kit (Ion 16S^TM^ Metagenomics Kit, Life Technologies; Carlsbad, CA, USA) and the Ion Torrent Personal Genome Machine (PGM) platform (Life Technologies, Carlsbad, CA, USA). Assessing and identifying bacterial species, microbial diversity, and performing grouping according to shared sequence characteristics is possible due to the high degree of conservation capacity of the 16S rRNA gene across bacterial domains. Taxonomical assignment is possible due to the presence of nine hypervariable regions (V1–V9) that contain sufficient sequence diversity to classify microbes. Furthermore, because conserved regions surround these variable regions, PCR amplification using universal primers is possible. To increase the resolving power of 16S rRNA profiling, primers in the Ion 16S^TM^ Metagenomics Kit were used to amplify variable regions 2, 4, and 8 in a single tube, resulting in amplicon fragments of approximately 250 base pairs (bp), 288 bp, and 295 bp, in length, respectively. In the second single tube, a multiplex PCR reaction targeted variable regions 3, 6 to 7, and 9, with resulting amplicon fragments of approximately 215 bp, 260 bp, and 209 bp, in length, respectively [13–16]. The primer set (V2-4-8, V3-5-6) composition followed Ion 16S™ Metagenomics Kit (Thermo Fischer Scientific). The primer pools were designed to target >80% of sequences found in the Greengenes database (>400,000 organisms curated) with 100% match for amplifying at least one variable region. After 16S rRNA PCR amplification, we loaded the PCR products on 1.5% agarose gel to confirm microbial DNA. Simultaneously, we loaded negative and positive controls to verify they did not contain non-microbial DNA. We have provided these data in a public repository at the following link: Data available from the Dryad Digital Repository: http://datadryad.org/review?doi=doi:10.5061/dryad.gj03f

Briefly, 100 ng of DNA was subjected to amplification of 16S rRNA libraries. Adaptors with barcode were ligated to the enzymatically end-repaired amplicons, purified using Agencourt AMPure XP beads (Beckman Coulter; Pasadena, CA, USA) and quantified using a bioanalyzer (2100 Bioanalyzer, Agilent Technologies; Santa Clara, CA, USA) according to the manufacturer’s protocol, and stored at -20°C until further processing. The library was diluted to 100 pM before template preparation. Template preparation of the barcoded libraries was performed using the Ion PGM Template OT2 400 Kit (Life Technologies; Carlsbad, CA, USA) and the Ion OneTouch 2 System (Life Technologies; Carlsbad, CA, USA). A maximum of 32 barcoded 16S samples were sequenced on an Ion 318 v2 chip (Life Technologies; Carlsbad, CA, USA) using the Ion PGM Sequencing 400 Kit (Life Technologies; Carlsbad, CA, USA) according to manufacturer’s instructions. After sequencing, the individual sequence reads were filtered by the PGM software to remove low-quality and polyclonal sequences. Sequences matching the PGM 3′ adaptor were also automatically trimmed. Subsequently, 16 rRNA sequences were analyzed using Ion Reporter Software (Life Technologies, Carlsbad, California, USA), which comprises a suite of bioinformatics tools that streamline and simplify analysis of semiconductor-based sequencing data.

### Data analysis

The data files were analyzed using QIIME software and default settings according to a tutorial for the microbiome study [17]. The reads were filtered and subjected to operational taxonomic unit (OTU) analysis (i.e., species grouping according to sequence) with a cut-off similarity of 97%. The OTU data were summarized into several OTU tables, using QIIME software for the evaluation of relative abundance of bacterial taxa [18].

Microbial alpha and beta diversity were estimated using QIIME software. Alpha diversity is defined as the diversity within a community and is measured by richness (S*), which estimates the number of species present; the Shannon diversity index (H) = $-\sum_{i=1}^{s}p_{i}ln(p_{i})$, where ***p***_***i***_ is proportion (n/N) of individuals of one particular species found (n), s is number of species; and the Simpson index (D) = $\frac{1}{\sum_{i=1}^{s}{p_{i}}^{2}}$, where ***p***_***i***_ is proportion (n/N) of individuals of one particular species found (n). The higher the score, the more diversity in the community. The Shannon diversity index and the Simpson index were used for assessing species diversity. The Shannon diversity index is an information statistic index and the Simpson index is a dominance index. The Mann-Whitney U test was used to identify differences between the two groups in richness (S^*^), Simpson index (D), and Shannon diversity index (H). R package software version 3.2.5 (R Foundation for Statistical Computing, Vienna, Austria) was used for alpha diversity analysis. Beta diversity is defined as the distance between communities, with distance estimated using principal coordinate analysis (PCoA) [17,18]. PCoA analysis assigns each sample to a position in a three-dimensional plot to reduce the multiple dimensions of OTU files using linear conversion formulas. Each PCoA plot was visualized in the three-dimensional structure, and the distances between the plots were summed. The total distances were compared within and between individuals with stable and exacerbated bronchiectasis. Beta-diversity was visualized using Bray-Curtis dissimilarity analysis.

## Results

### Demographics of patients with stable and acutely exacerbated bronchiectasis

A total of 17 patients were enrolled in this study; 9 had clinically stable bronchiectasis and 8 had acutely exacerbated bronchiectasis. Table 1 summarizes the demographic information of the patient pool. All 17 enrolled patients underwent BAL via bronchoscopy, and a BAL sample was obtained from each patient. Sputum samples were collected from all patients. In the DNA sequencing process, one BAL sample was excluded because the DNA was not sufficiently extracted; two BAL samples were excluded because the total number of reads was too low. Finally, 14 BAL samples were included in the analysis: 8 samples were obtained from patients with clinically stable bronchiectasis and 6 were collected from patients with acutely exacerbated bronchiectasis. The mean age of these 14 patients was 60 years (range 42–78 years) and 9 (64%) were female. Age and sex were not significantly different between the stable and exacerbated bronchiectasis groups.

**Table 1:** Demographics of stable and acutely exacerbated patients with non-CF bronchiectasis.

	Total N = 14	Stable group	Exacerbation group	*P*-value
		N = 8	N = 6	
Age, yr (range)	60.0 ± 8.5 (42–78)	58.8 ± 10.3	61.6 ± 5.8	0.568
Sex (Male), n (%)	5 (35.7%)	2 (25.0%)	3 (50%)	0.343
Bronchoalveolar lavage				
WBC count	1595.1 ± 2234.2	2036.6 ± 2652.1	1006.5 ± 1550.4	0.415
PMN (%)	68.9 ± 37.6	78.2 ± 32.7	56.5 ± 43.2	0.304
Lymphocyte cells (%)	6.6 ± 13.3	2.3 ± 2.4	12.3 ± 19.6	0.177

All data are expressed as mean ± SD except for Sex. **Abbreviations:** CF, cystic fibrosis; yr, year; n, number; PMN, polymorphonuclear cells; WBC, white blood cell

The cause(s) of bronchiectasis were unknown in most patients. One patient acquired measles in childhood and another had focal atelectasis. None of patients had been treated for pulmonary tuberculosis. Common comorbid diseases included chronic obstructive pulmonary disease (n = 2) and diabetes (n = 2). Although there were no current smokers, two patients were previous smokers. BAL fluid analysis showed no significant differences in white blood cell count and differential cell count between the two groups.

### Comparison of isolated organisms between microbiological and molecular tests

Table 2 lists the organisms identified by BAL quantitative culture, sputum culture, and 16S rRNA sequencing. There were several differences in the type of organisms identified using conventional microbiological and molecular tests. BAL quantitative culture revealed only dominant organisms, while molecular methods using 16S rRNA sequencing provided previously unknown information. We described the three most abundant bacteria in each of the samples. In the BAL quantitative and sputum cultures, *P aeruginosa* (5 patients) and *H influenzae* (4 patients) were the major organisms. The others were *Klebsiella pneumoniae* (2 patients) and *S aureus* (1 patient). The causative organism in 2 patient samples could not be identified. BAL cultures identified causative organisms in 11 patients; however, sputum cultures identified organisms in only 3. 16S rRNA sequencing identified several potentially causative organisms in all patients. There were discrepancies between microbiological and molecular tests in 3 samples (ST7, EX3 and EX6). 16S rRNA sequencing identified potentially causative organisms in undetermined samples tested using microbiological tests of BAL and sputum (ST2 and ST6). When *H influenzae* was identified as a causative organism, *Haemophilus aegyptius* was present in all cases (n = 7). An anaerobic bacterial culture process was not used for microbiological testing; thus, anaerobes could not be isolated. However, 16S rRNA gene sequencing revealed several clinically important anaerobic bacterial species including *Prevotella* (3 patients), *Veillonella* (1 patient), *Porphyromonas* (1 patient), and *Tannerella* (1 patient).

**Table 2:** Bacteria isolated from BAL quantitative cultures, sputum cultures, and 16S rRNA sequencing by BAL and sputum samples.

BAL ID	Age	Sex	Clinical status	BAL culture	Sputum culture	16S rRNA sequencing		
						Primary	Secondary	Tertiary
ST1	59	F	Stable	*Pseudomonas aeruginosa*	*α-streptococcus*	*Haemophilus_aegyptius*	*Haemophilus_influenzae*	*Pseudomonas_aeruginosa*
ST2	42	F	Stable	*α-streptococcus*	*-*	*Haemophilus_aegyptius*	*Haemophilus_influenzae*	*Prevotella_melaninogenica*
ST3	58	F	Stable	*-*	*Pseudomonas aeruginosa*	*Pseudomonas_aeruginosa*	*Pseudomonas_mendocina*	*Pseudomonas_sp*.
ST4	78	M	Stable	*Haemophilus influenzae*	*-*	*Haemophilus_aegyptius*	*Haemophilus_influenzae*	*Veillonella_atypica*
ST5	53	F	Stable	*Staphylococcus aureus*	*Staphylococcus aureus*	*Prevotella_melaninogenica*	*Staphylococcus_aureus*	*Neisseria_subflava*
ST6	54	M	Stable	*-*	*α-streptococcus*	*Moraxella_catarrhalis*	*Porphyromonas_gingivalis*	*Tannerella_forsythia*
ST7	62	F	Stable	*Pseudomonas aeruginosa*	*α-streptococcus*	*Prevotella_pallens*	*Staphylococcus_warneri*	*Neisseria_subflava*
ST8	65	F	Stable	*Haemophilus influenzae*	*-*	*Haemophilus_influenzae*	*Haemophilus_aegyptius*	*Rothia_mucilaginosa*
EX1	64	M	Exacerbated	*Pseudomonas aeruginosa*	*α-streptococcus*	*Pseudomonas aeruginosa*	*Pseudomonas_sp*.	*Pseudomonas_mendocina*
EX2	63	F	Exacerbated	*Haemophilus influenzae*	*α-streptococcus*	*Haemophilus_aegyptius*	*Haemophilus_influenzae*	*Prevotella_melaninogenica*
EX3	64	F	Exacerbated	*Haemophilus influenzae*	*α-streptococcus*	*Escherichia_coli*	*Cronobacter_sakazakii*	*Escherichia_vulneris*
EX4	69	M	Exacerbated	*Pseudomonas aeruginosa*	*-*	*Haemophilus_aegyptius*	*Pseudomonas_aeruginosa*	*Haemophilus_influenzae*
EX5	52	F	Exacerbated	*Klebsiella pneumoniae*	*-*	*Klebsiella_pneumoniae*	*Prevotella_melaninogenica*	*Neisseria_perflava*
EX6	58	M	Exacerbated	*Klebsiella pneumoniae*	*Klebsiella pneumoniae*	*Haemophilus_aegyptius*	*Haemophilus_influenzae*	*Haemophilus_haemolyticus*

All identified organisms are described by species, counted lesser than 0.01% compared to primary organisms were discarded. **Abbreviations:** BAL; bronchoalveolar lavage, rRNA; ribosomal ribonucleic acid, ST; stable bronchiectasis, EX; exacerbated bronchiectasis

### Number of observed bacterial 16S rRNA gene sequences

Table 3 summarizes the sequencing data, which contained approximately 7.5 million reads in total, with an average of 534,857 reads per valid sample. Two samples were excluded because the total number of reads was too small to analyze. Table 4 lists the relative abundance of taxa at the genus level from the sequence pool of stable and exacerbated bronchiectasis. In the stable bronchiectasis group, *Haemophilus* (42.4%), *Pseudomonas* (22.2%), *Moraxella* (17.1%), *Prevotella* (4.3%), *Staphylococcus* (3.9%), and *Streptococcus* (1.4%) were relatively abundant. In the exacerbation group, *Haemophilus* (43.1%), *Pseudomonas* (24.0%), *Klebsiella* (12.5%), *Prevotella* (4.6%), *Roseomonas* (2.4%), and *Aeromonas* (1.8%) were abundant. Both groups showed similar compositions. Furthermore, of the 167 OTUs, nine accounted for >90% of the total sequences generated, indicating that the majority of the observed community richness resulted from low abundance taxa.

**Table 3:** Number of observed bacterial sequences from 14 BAL samples.

BAL ID	Total number of reads	Number of valid reads	Number of reads ignored	Mapped reads in sample	Un-Mapped reads
ST1	585,459	409,730	64,458	345,272	0
ST2	831,240	537,327	80,600	456,717	10
ST3	1002,313	796,314	67,293	729,021	0
ST4	585,459	409,730	64,458	345,272	0
ST5	565,943	350,416	102,157	248,146	113
ST6	1099,214	715,728	139,722	575,938	68
ST7	469,296	332,839	81,219	251,578	42
ST8	1273,937	834,926	105,488	729,427	11
EX1	844,917	528,643	80,248	447,814	581
EX2	318,360	213,047	39,690	173,051	306
EX3	804,446	479,406	80,406	398,985	15
EX4	148,527	104,477	26,202	78,275	0
EX5	1909,570	1104,952	259,329	843,487	2,136
EX6	1327,923	670,465	93,748	576,717	0
Total	11766,604	7488,000	1285,018	6199,700	3,282
Average	840,471	534,857	91,787	442,835	234

**Abbreviations:** BAL; bronchoalveolar lavage, rRNA; ribosomal RNA, ST; stable bronchiectasis, EX; exacerbated bronchiectasis

**Table 4:** Relative abundance and distribution from the sequence pool for the top 30 taxon groups from BAL samples in clinically stable and exacerbated bronchiectasis.

	Stable			Exacerbation		
order	genus	frequency	Proportion (%)	genus	frequency	Proportion (%)
1	*Haemophilus*	1,216,187	42.42	*Haemophilus*	584,362	43.13
2	*Pseudomonas*	635,524	22.17	*Pseudomonas*	324,463	23.95
3	*Moraxella*	491,222	17.13	*Klebsiella*	169,204	12.49
4	*Prevotella*	123,937	4.32	*Prevotella*	62,653	4.62
5	*Staphylococcus*	111,237	3.88	*Roseomonas*	31,822	2.35
6	*Streptococcus*	39,269	1.37	*Aeromonas*	24,837	1.83
7	*Campylobacter*	32,833	1.15	*Campylobacter*	16,247	1.20
8	*Comamonas*	31,027	1.08	*Neisseria*	12,945	0.96
9	*Neisseria*	29,208	1.02	*Streptococcus*	10,487	0.77
10	*Gallibacterium*	20,846	0.73	*Selenomonas*	10,336	0.76
11	*Porphyromonas*	17,546	0.61	*Gallibacterium*	8,529	0.63
12	*Veillonella*	12,841	0.45	*Veillonella*	8,267	0.61
13	*Roseomonas*	12,274	0.43	*Rothia*	8,194	0.60
14	*Fusobacterium*	7,696	0.27	*Serratia*	6,839	0.50
15	*Selenomonas*	6,796	0.24	*Porphyromonas*	6,688	0.49
16	*Corynebacterium*	6,256	0.22	*Peptostreptococcus*	5,843	0.43
17	*Tannerella*	5,259	0.18	*Propionibacterium*	5,030	0.37
18	*Lautropia*	4,367	0.15	*Micrococcus*	4,405	0.33
19	*Klebsiella*	4,338	0.15	*Fusobacterium*	3,296	0.24
20	*Serratia*	4,025	0.14	*Paracoccus*	2,802	0.21
21	*Micrococcus*	3,892	0.14	*Lactobacillus*	2,794	0.21
22	*Gordonia*	3,210	0.11	*Escherichia*	2,624	0.19
23	*Oribacterium*	3,193	0.11	*Mycobacterium*	2,349	0.17
24	*Peptostreptococcus*	3,050	0.11	*Staphylococcus*	2,248	0.17
25	*Capnocytophaga*	1,954	0.07	*Sphingomonas*	2,184	0.16
26	*Achromobacter*	1,898	0.07	*Catonella*	1,874	0.14
27	*Dialister*	1,803	0.06	*Gordonia*	1,736	0.13
28	*Acinetobacter*	1,760	0.06	*Oribacterium*	1,664	0.12
29	*Eubacterium*	1,750	0.06	*Butyrivibrio*	1,641	0.12
30	*Actinobacillus*	1,615	0.06	*Eubacterium*	1,497	0.11

**Abbreviation:** BAL; bronchoalveolar lavage

### Community compositions of the major identified bacterial genus in pooled sequences

**Fig 1:**
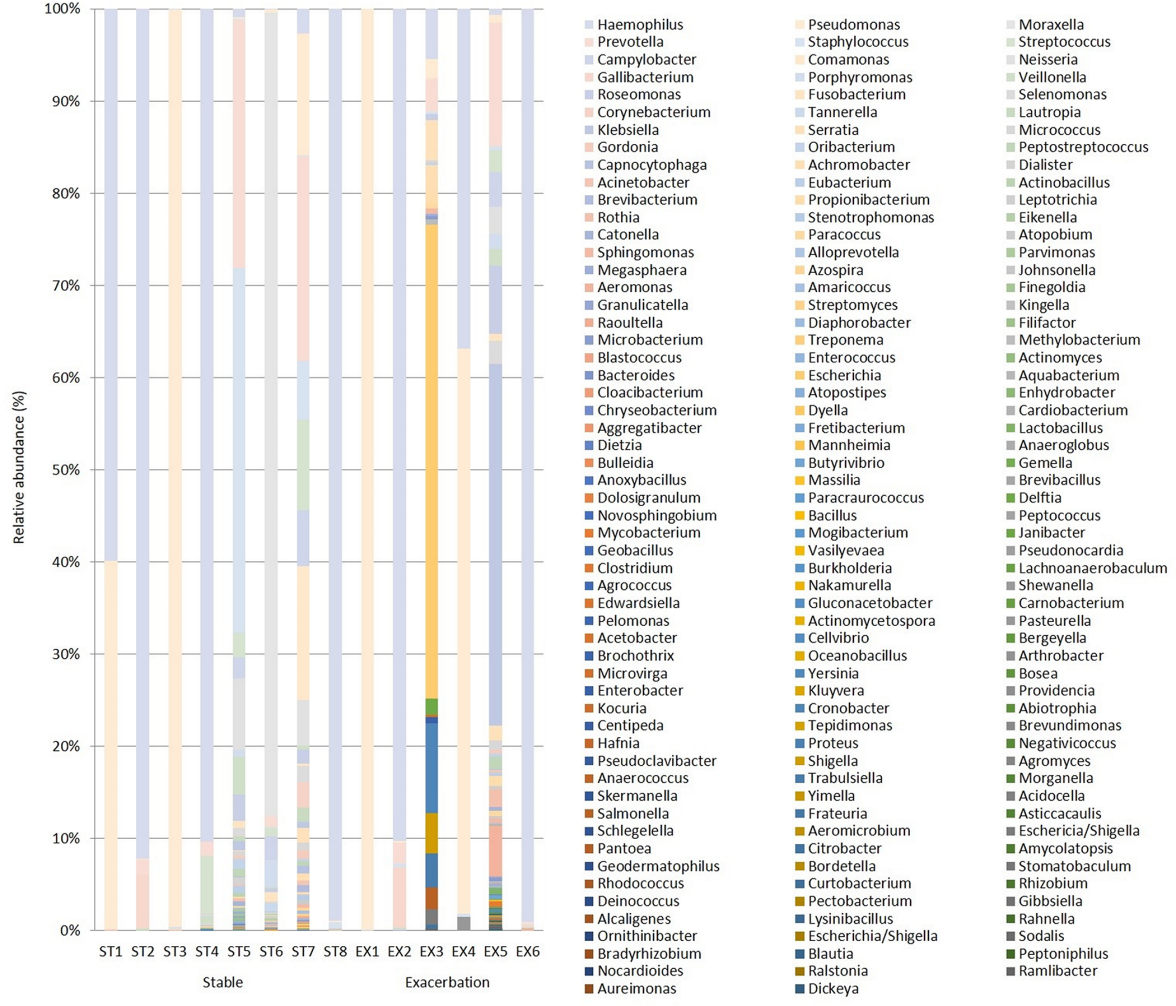
Relative abundance of taxa in bronchoalveolar lavage (BAL) samples from patients with stable and exacerbated bronchiectasis according to 16S ribosomal RNA gene sequencing (genus level).

**Fig 2:**
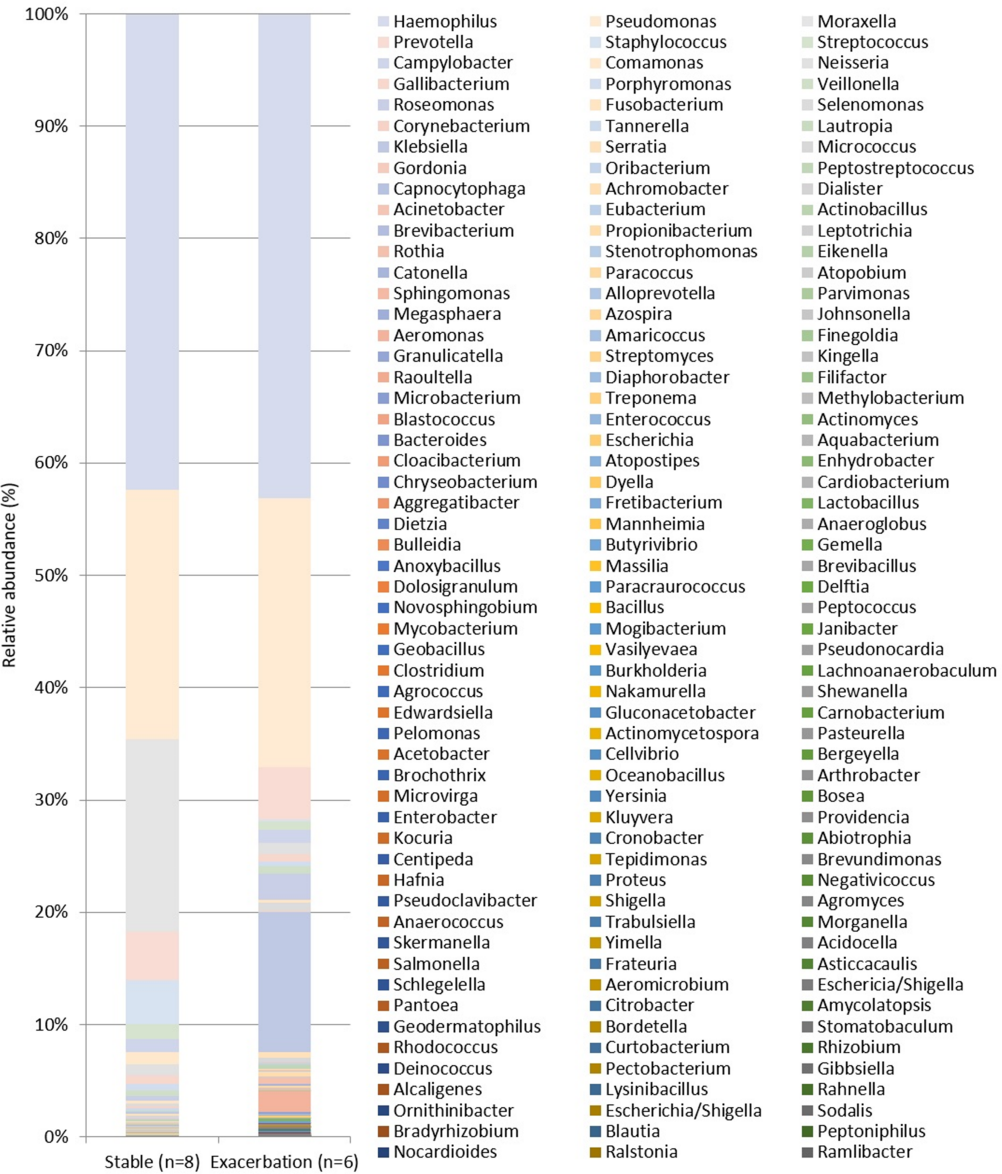
Relative abundance of taxa in pooled sequences of patients with stable and exacerbated bronchiectasis groups according to 16S ribosomal RNA gene sequencing (genus level). The alpha diversity index was estimated to compare organism richness and the diversity between two groups using two diversity indices: the Shannon diversity index (H) and the Simpson’s index (D).

Fig 1 shows the relative abundance of community composition from identified bacterial genus in each patient with stable bronchiectasis (n = 8) and exacerbated bronchiectasis (n = 6). The pooled community composition between stable (n = 8) and exacerbated (n = 6) bronchiectasis was compared to determine whether clinical status affected community composition. Fig 2 shows similar patterns of genus and family distribution in both groups. Similar patterns of genus distribution were observed in the stable and exacerbated bronchiectasis groups.

**Fig 3:**
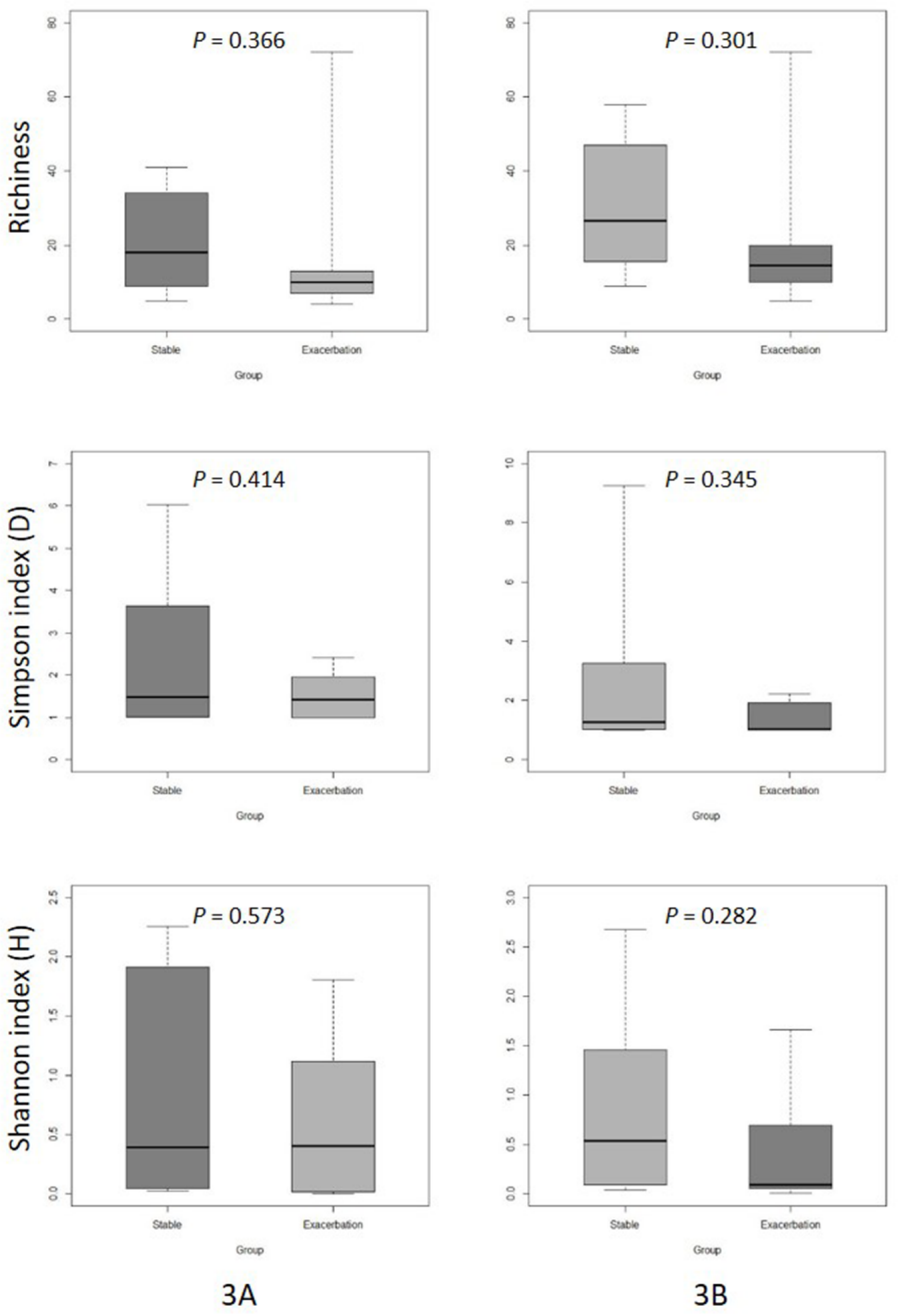
**Boxplot comparison of mean diversity in BAL samples between clinically stable and exacerbated bronchiectasis patients (3A: genus, 3B: family).** Three measures are presented: upper (richness), middle (Simpson’s index), and lower (Shannon diversity index). The top and bottom boundaries of each box indicate the 75th and 25th quartile values, respectively. Lines within each box represent the 50th quartile values. Ends of whiskers mark the lowest and highest diversity values in each box.

There was no significant difference in microbial community diversity between the two groups (Fig 3).

### Comparison of microbial community structure between stable and exacerbated bronchiectasis groups

**Fig 4:**
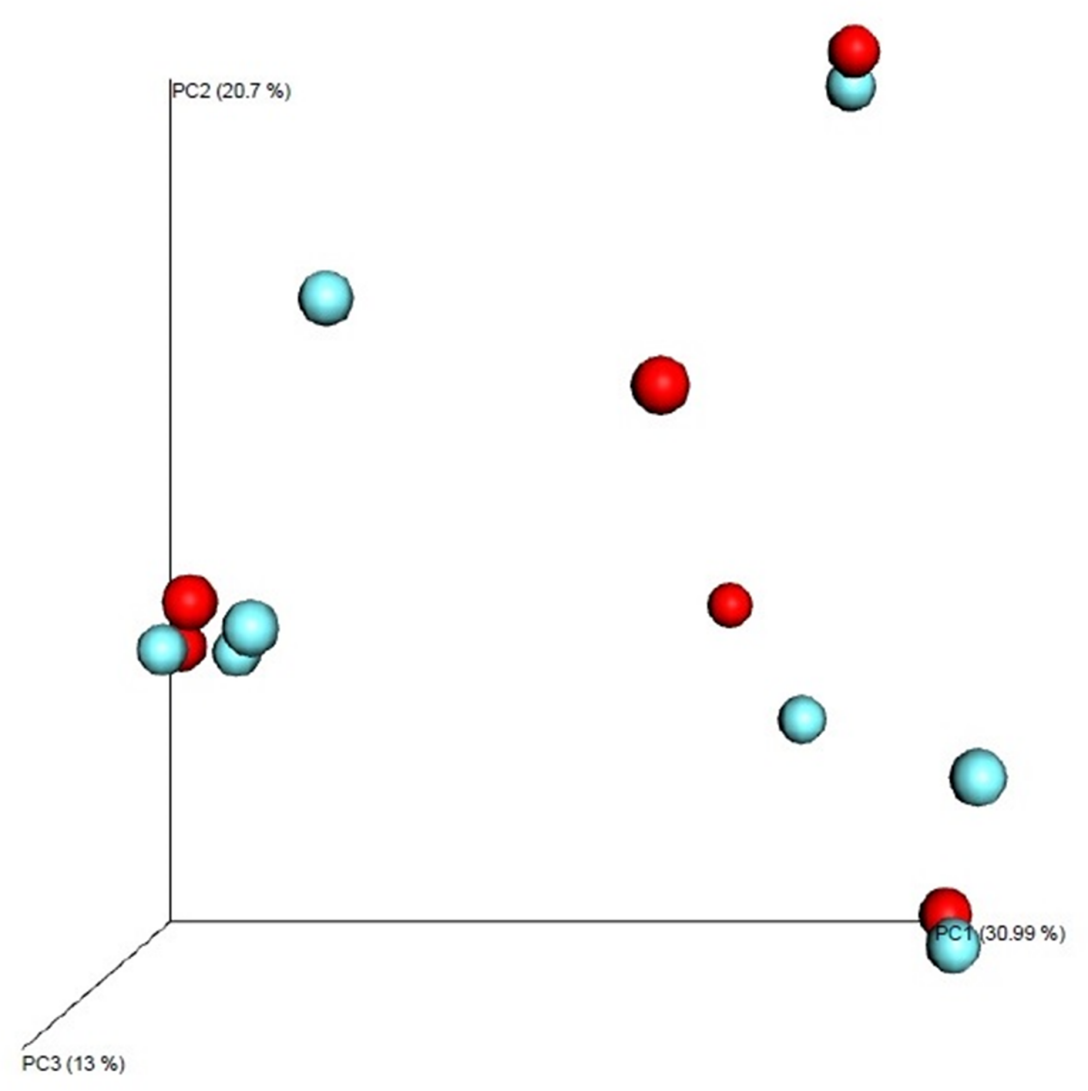
PCoA plots of microbial communities in patients with stable and exacerbated bronchiectasis. The blue spheres indicate patients with stable bronchiectasis and red spheres indicate patients with exacerbated bronchiectasis.

**Fig 5:**
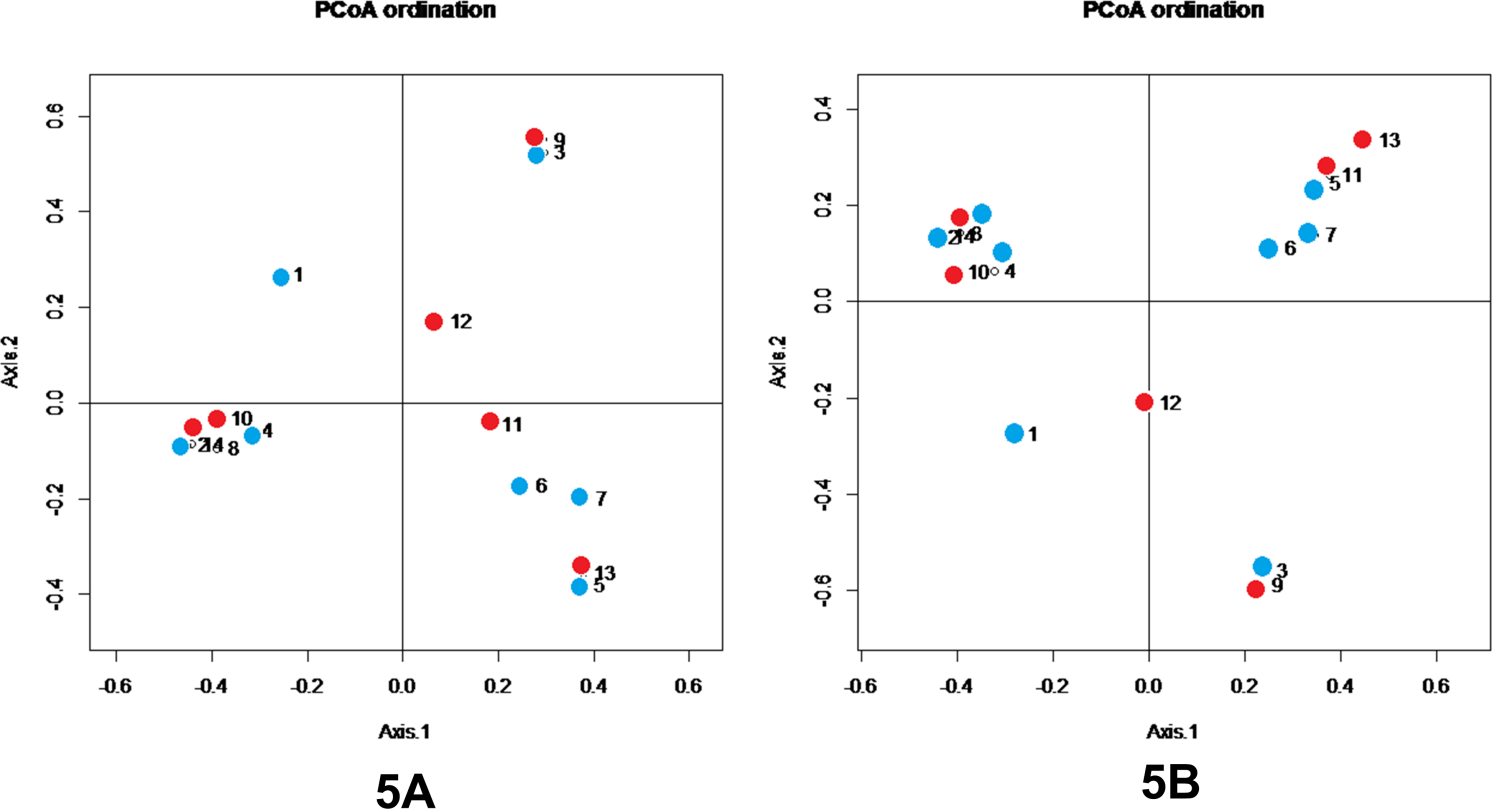
**Two-dimensional PCoA plot of microbial communities in patients with stable and exacerbated bronchiectasis (5A: genus, 5B: family).** Axes show percentage of contribution, Axis 1: 25.86%, Axis 2: 17.53% Stable bronchiectasis (blue circles: 1–8), exacerbated bronchiectasis (red circles: 9–14)

To estimate the distance between communities, beta diversity was analyzed using PCoA plots. Fig 4 shows the Bray-Curtis metrics, visualized as a 3-dimensional PCoA plot. Beta diversity between stable bronchiectasis and exacerbated bronchiectasis patients was estimated using PCoA plots generated by the QIIME software with linear conversion formulas. Three-dimensional PCoA plots were visualized, in which the 3 axes and each contribution ratio (principal coordinate, PC 1–3, %) were depicted. There were no definite differences between the 8 stable bronchiectasis communities and 6 exacerbated bronchiectasis communities (Fig 4). A two-dimensional PCoA plot is shown using Bray-Curtis dissimilarity in Fig 5. Points 1 to 8 refer to stable bronchiectasis patients, and points 9 to 14 refer to exacerbated bronchiectasis patients. There was no clustering between the stable and exacerbated groups. Samples showing close clustering tend to gather according to the dominant bacteria, rather than clinical status.

## Discussion

In this study, we used microbiological culture techniques and culture-independent next-generation sequencing to perform a comprehensive analysis of lung microbiome composition in patients with clinically stable and acutely infected exacerbated bronchiectasis. Previous studies have investigated stable bronchiectasis using aerobic bacterial cultures. In this study, bacterial culture identified *P aeruginosa*, *H influenzae*, *K pneumoniae*, *and S aureus* as the major organisms and species, with rates similar to those reported in previous studies [19–21]. In contrast, culture-independent 16S rRNA gene sequencing provided a more comprehensive characterization of lung microbial community composition. Two bacterial organisms that could not be identified using BAL quantitative cultures and sputum cultures (*M catarrhalis* and *H influenzae*) were identified using DNA sequencing methods. *M catarrhalis* is a common major pathogen identified in non-CF bronchiectasis [4,19,20]. Using culture-independent molecular techniques, *M catarrhalis* was the third most abundant (11.6%) bacteria in the observed taxa from the sequence pool of 14 BAL samples, in contrast to the results from microbiological cultures. Furthermore, DNA sequencing revealed potentially pathogenic anaerobic organisms, such as *Prevotella* and *Veillonella* species, in BAL samples, which are exceedingly difficult to isolate using routine aerobic culture methods. Recent studies using molecular-based techniques have reported that anaerobic bacteria are commonly found and abundant in lower airway samples from patients with CF and non-CF bronchiectasis [22,23]. Nevertheless, the role of anaerobic species in the pathophysiology of CF and non-CF bronchiectasis remains unclear. Tunney et al. [18] suggested that an increase in anaerobic load relative to aerobic load may be a contributing factor to the onset of exacerbation of bronchiectasis.

16S rRNA gene sequencing analysis revealed relative abundance of taxa at the genus level from the sequence pool of stable and exacerbated bronchiectasis. Both groups showed similar relative abundance and distribution of taxa (Fig 2). There were no differences between stable and exacerbated bronchiectasis groups (Fig 3). Our results suggest that microbial composition in the lung can be partitioned into two groups, similar to the observations in CF and non-CF bronchiectasis [24–27]. One group is composed of a relatively small number of dominant taxa in that environment: in our stable and exacerbated bronchiectasis groups, *H influenzae*, *P aeruginosa*, *and S aureus* were dominant. The other group consisted of less common taxa in low abundance, which accounts for most of the observed community richness. Individual patients have unique microorganisms, and the composition of the airway community varies from patient to patient. In our study, patient-specific microbial communities were dominated by one or several genera and were maintained over time, resulting in distinctive individual profiles. This finding is consistent with that in studies investigating lung microbiome in other respiratory diseases [10,24].

Our intent was to provide a better understanding of the pathogenesis of acute exacerbation in bronchiectasis. Acute exacerbations are the most important cause of morbidity in patients with bronchiectasis and are associated with disease progression [28,29]. To date, however, the exact cause(s) of exacerbation remain unclear. Possible hypotheses include changes in airway bacterial community composition, emergence of new species or strains, or the spread of the same species to new regions in the lung. Previous studies investigating chronic obstructive pulmonary disease suggested that the acquisition of new stains of *H influenzae or P aeruginosa* is associated with occurrence of exacerbation [30,31]. We found no differences in lung microbial community composition between patients with exacerbated bronchiectasis compared with stable bronchiectasis. In the alpha diversity analysis, microbial community diversity in patients with stable bronchiectasis suggested a tendency toward more diverse genera than that in patients with exacerbated bronchiectasis, although without significant differences. Beta diversity analysis revealed no similarity of microbial communities within groups (stable or exacerbated).

This study, however, had several limitations, the first of which was the small number of patients, which in turn resulted in low statistical power to detect differences. We collected BAL samples via bronchoscopy, not sputum or induced sputum and, given that BAL via bronchoscopy is an invasive procedure compared with sputum collection, enrolling a large number of patients was impossible. Nevertheless, molecular analysis of BAL samples from bronchiectasis patients represents one strength of our study. Although BAL is an invasive procedure, it does not contain oral flora and, consequently, is a particularly accurate reflection of the lung microbiota. Based on the results of this exploratory study, we will suggest directions for future studies. Second, we could not collect BAL samples of clinically stable and exacerbated from same patients, because BAL via bronchoscopy is an invasive procedure. Third, acute exacerbation of bronchiectasis could not be clearly differentiated from stable bronchiectasis. Fuchs et al. [12] defined criteria including 12 signs or symptoms; however, patients with clinically stable bronchiectasis usually experience symptoms daily. BAL fluid analysis in our study showed discrepancies in the stable and exacerbated bronchiectasis groups. Fourth, we performed agarose gel (1.5%) electrophoresis for our presumptive negative control; however, the absence of a band does not necessarily mean the absence of an amplified product. DNA sequencing would have been a more stringent negative control. Two BAL samples were excluded from the analysis because the total number of reads was too low. However, we could identify bands representing these two samples in the gel results. Fifth, next-generation sequencing 16S rRNA metagenomic analysis using a multiplex targeted PCR method is very sensitive to lower abundant species, and data analysis is relatively easy and compact. It still, however, has the limitation of primer binding sites only for known sequences. Salipante et al. compared the performances of two common sequencing platforms, Illumina MiSeq and Ion Torrent PGM, for bacterial community profiling using 16S rRNA (V1-V2) amplicon sequencing, and reported that a higher rate of sequencing errors was observed using the Ion Torrent platform; however, the absolute difference in error rates between the two platforms was not significant [32]. The Ion 16S^TM^ Metagenomics Kit used in our study simultaneously examines 7 of the 9 hypervariable regions in the 16S rRNA gene. This kit is well-validated to detect bacterial strains in the microbial mock community standard material HM-277D (BEI resources) and supporting publications [13–16]. Nonetheless, we cannot exclude the possibility that a few bacterial variable regions could be underestimated or fail to be detected due to mismatched primer binding sites in the 16S variable regions.

## Conclusions

*H influenzae*, *P aeruginosa and M catarrhalis*, *and Prevotella* species were the major organisms and species identified, and were not different according to the clinical status of patients with stable or exacerbated bronchiectasis. Although culture-independent 16S rRNA gene sequencing provided more comprehensive characterization of lung microbial community composition, we found no significant differences between the lung microbiome of patients with clinically stable and exacerbated bronchiectasis. In our study, patient-specific microbial communities were dominated by one or several genera, regardless of clinical status, which is stable or acutely exacerbated. DNA sequencing could identify potentially pathogenic organisms unable to be identified with microbiological methods.

## Supporting information

S1 File DNA extraction. DNA extraction protocol.
(DOCX)
