# Supplementary material for: Differences of lung microbiome in patients with clinically stable and exacerbated bronchiectasis
Source: PLoS One. 2017 Aug 22;12(8):e0183553. doi: 10.1371/journal.pone.0183553 (PMC5567645; doi:10.1371/journal.pone.0183553)
Supplement: S1 File — DNA extraction protocol. (DOCX) [file pone.0183553.s001.docx]

**DNA extraction**

DNA was extracted using MG blood Genomic DNA Extraction SV kit protocol (MGmed; Seoul, Korea) according to the manufacturer’s protocol. Briefly, a BAL sample was contained on dry ice, transferred to an Eppendorf tube, and weighted. Approximately 20 ml of BAL sample was vortexed thoroughly until homogenization was achieved. 20mg/ml of proteinase K solution provided into the bottom of a 1.5 ml tube. 200 μL of sample was transferred to the tube. To acquire RNA-free DNA, added 20 μL of RNase solution to the sample, pipet 2~3 times to mix and incubate for 2 minute at room temperature. Added 200 μL of cell lysis buffer BL to the tube. Vortex the tube to mix thoroughly. Incubated at 56℃ for 10 minutes. Spin down briefly to remove any drops from inside of the lid. Added 200 μL of absolute ethanol to the sample, pulse-vortex to mix the sample thoroughly, and spin down briefly to remove any drops from inside of the lid. Transferred the mixture to the MG SV column carefully, centrifuged for 1 minute at 6,000 * g above (>8,000 rpm), and replaced the collection tube with new one. Added 600 μL of wash buffer BW, centrifuged for 1 minute at 6,000 * g (8,000 rpm) and replaced the collection tube with new one. Applied 700 μL of wash buffer TW. Centrifuged for 1 minute at 6,000 * g above (>8,000 rpm). Discarded the pass-through and reinsert the MG SV column back into the collection tube. Centrifuged at full speed (12,000 * g, at least) for 1 minute to remove residual wash buffer. Placed the MG SV column in a fresh 1.5 ml tube. Ensure that sterilized water is dispensed directly onto the center of MG SV column membrane for optimal elution of DNA. Added 200 μL of distilled deionized water (> pH 7.0). Incubated for 1 minute at room temperature. Centrifuged at full speed. All DNA samples were stored at -20°C until use for PCR reactions.
